# Preparation and Application of Co-Doped Zinc Oxide: A Review

**DOI:** 10.3390/molecules29143373

**Published:** 2024-07-18

**Authors:** Zhaoyu Luo, Ping Rong, Zhiyuan Yang, Jianhua Zhang, Xiangyu Zou, Qi Yu

**Affiliations:** Shaanxi Laboratory of Catalysis, School of Materials Science and Engineering, Shaanxi University of Technology, Hanzhong 723001, China; luozhaoyu1998@163.com (Z.L.); rongping1994@163.com (P.R.); 17835696667@163.com (Z.Y.); zhangts12@foxmail.com (J.Z.); zou800403@163.com (X.Z.)

**Keywords:** ZnO, co-doping, preparation, application

## Abstract

Due to a wide band gap and large exciton binding energy, zinc oxide (ZnO) is currently receiving much attention in various areas, and can be prepared in various forms including nanorods, nanowires, nanoflowers, and so on. The reliability of ZnO produced by a single dopant is unstable, which in turn promotes the development of co-doping techniques. Co-doping is a very promising technique to effectively modulate the optical, electrical, magnetic, and photocatalytic properties of ZnO, as well as the ability to form various structures. In this paper, the important advances in co-doped ZnO nanomaterials are summarized, as well as the preparation of co-doped ZnO nanomaterials by using different methods, including hydrothermal, solvothermal, sol-gel, and acoustic chemistry. In addition, the wide range of applications of co-doped ZnO nanomaterials in photocatalysis, solar cells, gas sensors, and biomedicine are discussed. Finally, the challenges and future prospects in the field of co-doped ZnO nanomaterials are also elucidated.

## 1. Introduction

As human society has entered industrial civilization, the development model has become increasingly highly dependent on the input of fossil energy and material resources, which has generated numerous ecological and environmental problems, thereby leading to global climate change. Carbon neutrality was proposed at the 75th General Debate of the United Nations General Assembly to actively address the major global challenge of climate change. As a result, there is growing concern about the ecological environment and issues such as water pollution.

At present, semiconductor oxides such as ZnO [1], TiO_2_ [2], SnO_2_ [3], Fe_2_O_3_ [4], WO_3_ [5], and In_2_O_3_ [6] have received a lot of attention from researchers because of their high photocatalytic efficiency [7]. Among them, ZnO, as a broad-band-gap semiconductor material, has been favored by many researchers for its wide band gap (3.37 eV), large exciton binding energy (60 meV), and non-toxicity and non-hazardous properties [8]. However, the rapid complexation of photogenerated electron-and-hole pairs of ZnO limits its intensive application in photocatalysis, as well as in other fields including solar cells, gas sensors, optoelectronic devices, and biomedicine [9]. After continuous research, it has been found that doping is one of the best methods to promote carrier separation and thus improve the performance of ZnO nanostructures [10]. This is principally because doping has three effects: (i) narrowing the band gap and promoting adsorption; (ii) improving the electrical conductivity and carrier mobility of ZnO; and (iii) changing the conduction band (CB) position and valence band (VB) of ZnO [11]. Especially, the co-doping technique is a very promising strategy to effectively tune the optical, electrical, magnetic, and photocatalytic properties of ZnO, which has become a current research hotspot. The incorporation of a single dopant produces ZnO with unstable reliability, thus facilitating the study of co-doping techniques [12]. Currently, there are many studies on the optical, electrical, and photocatalytic properties of co-doped ZnO. For example, Petronela Pascariu et al. [13] synthesized Ni-Co-doped ZnO nanoparticles with enhanced photocatalytic activity using co-precipitation method. The photocatalytic degradation activity of rhodamine B (RhB) was enhanced at Ni-Co doping levels of ~0.2%. O.F. Kolomys et al. [14] used the light furnace method to grow co-doped ZnO particles. Optical and structural properties of co-doped ZnO particles were systematically investigated using optical absorption spectroscopy. In addition, co-doped ZnO has good ferromagnetism due to the fact that the unpaired electrons in the d or f states of transition or rare earth metals can induce magnetism in non-magnetic semiconductors. Hu et al. [15] demonstrated that the magnetic properties were transformed from paramagnetic to room temperature ferromagnetic upon the addition of Zn_0.98_Co_0.02_O by co-doping at 2%. First-principle calculations further showed that the strong hybridization between the Co three-dimensional state and the Cu-induced donor impurity band at the Fermi energy level effectively enhances the indirect ferromagnetic superexchange between Co ions and is responsible for the emergence of ferromagnetism in Co-Cu-co-doped ZnO. In addition, the energy preference orientation of ZnO along the (001) direction (the c-axis of the fibrillated ZnO structure) originates from the lowest surface energy in the (002) plane. The c-axis growth direction is perpendicular to the substrate surface, and the piezoelectric properties of the film along this direction can be used in acoustic wave devices [16]. For instance, the Ni-V-co-doped ZnO prepared by the hydrothermal method by Kiruthika Ramany et al. [17] has a greatly reduced internal resistance, resulting in a better conducting p-n junction, which can be used to fabricate an enhanced self-powered ZnO piezoelectric accelerometer with significant sensitivity for applications in nano-electromechanical system accelerometers. ZnO is a promising semiconductor material, but despite breakthroughs in some areas, it has not yet been commercialized due to some co-doping technology gaps and economic constraints. However, both challenges and opportunities exist, and key factors such as the further improvement and refinement of co-doping techniques, new preparation techniques, accurate characterization techniques, and advanced computational simulations still need to be further investigated.

**Figure 1:**
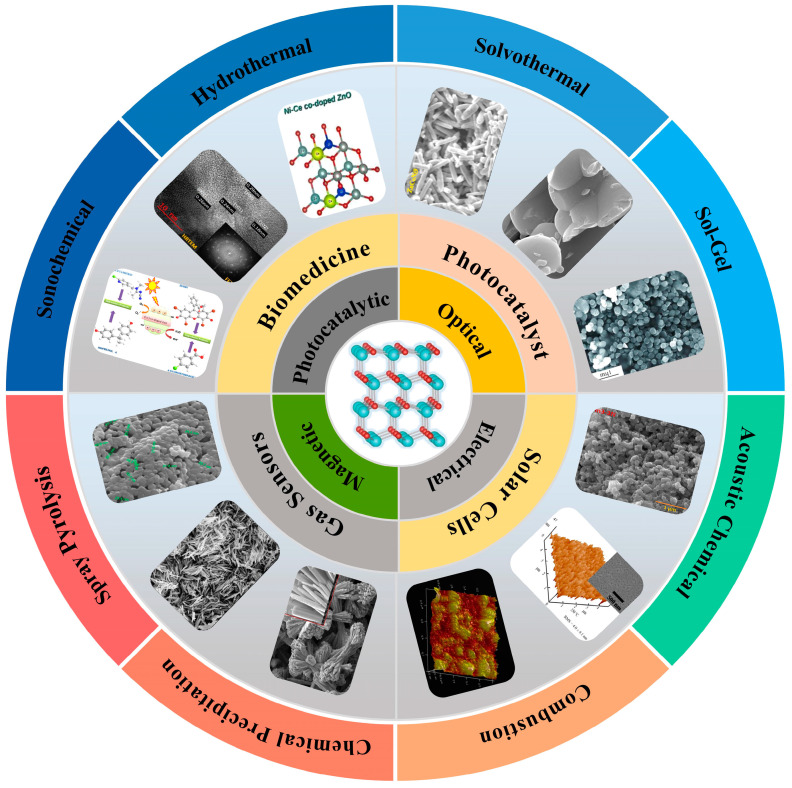
Schematic diagram of recent advances in the preparation, properties, and applications of co-doped ZnO nanomaterials [18,19,20,21,22,23,24,25,26,27,28,29].

In this paper, the advances of co-doped ZnO nanomaterials have been summarized in detail, focusing on the various preparation methods of co-doped ZnO nanomaterials. In addition, the applications in photocatalysis, solar cells, gas sensors, and biomedicine are also discussed. Meanwhile, the challenges and prospects facing the field of co-doped ZnO nanomaterials are elucidated (Figure 1).

## 2. Preparation Methods of Co-Doped ZnO

In general, the physical characteristics and applications of co-doped ZnO depends on factors such as particle size, impurity, morphology, band gap, number of surface-active sites, dopant type, and dopant concentration [10,11], which in turn are related to its preparation methods. In order to continually explore the applications of co-doped ZnO, various methods have been developed to prepare co-doped ZnO nanocomposites, including hydrothermal [17,30,31,32,33], solvothermal [34], sol-gel [35,36], combustion [37], spray pyrolysis [38], wet chemical “liquid ceramics” [39], chemical [40], radio frequency (RF) magnetron sputtering [8,41], acoustic chemistry [42], and electron beam evaporation deposition [43]. Different types of morphologies and nanostructures can be obtained using different synthesis methods. These nanostructures include thin films, nanoplates, nanospheres, nanowires, nanorods, nanotubes, nanoflowers, nanofibers, and nanoribbons [44,45,46,47,48,49].

### 2.1. Hydrothermal

The hydrothermal method is a simple and well-established synthetic technique that mimics the growth of crystals during natural mineralization, using water as a solvent and reacting in a closed system at a certain temperature and pressure. It is considered to be one of the most attractive methods for the preparation of ZnO due to its ease of set-up, controllability, cost-effectiveness, relatively low temperature, and environmental friendliness. Moreover, the hydrothermal method can be used to obtain different forms of co-doped ZnO by adjusting parameters such as the reagent concentration, temperature, reaction time, pH, additives, and surfactants [50,51,52,53,54,55,56]. To date, hydrothermal synthesis, as an important preparation technique, has been extensively used to prepare co-doped ZnO systems. It has the potential to be studied both in the degradation of organic dyes and in the magnetic studies of co-doped ZnO, among others.

Yang et al. [57] prepared Zn_1−2x_Fe_x_Ce_x_O (x = 0, 0.01, 0.03, 0.04) composites; Fe(NO_3_)_3_·9H_2_O, Ce(NO_3_)_3_·6H_2_O, and LiOH·H_2_O were used as the Fe, Ce, and base sources, respectively. Varying ratios of Fe(NO_3_)_3_·9H_2_O and Ce(NO_3_)_3_·6H_2_O (0, 0.03, 0.09, and 0.12 mmol) and 3 mmol of Zn(NO_3_)_2_·6H_2_O were added to 50 mL of deionized water and stirred for 1 h. Then, 0.004 g of hexadecyl trimethyl ammonium bromide (CTAB) was added as a surfactant and stirring was continued for 2 h to mix the reagents uniformly. The mixed solution was poured into a Teflon-lined stainless steel autoclave of 100 mL capacity and heated at 150 °C for 2 h. After natural cooling, the sample was removed, washed with deionized water and pure ethanol 3 times, and placed in a vacuum-drying oven at 80 °C for 8 h. After cooling, the sample was ground, placed in a muffle furnace, and sintered at 600 °C for 2.5 h to obtain the final sample, and then the photocatalytic efficiency of Fe-Ce-co-doped ZnO on the organic dye methylene blue under simulated sunlight was investigated. Moreover, Santanu Das et al. [58] reported the presence of ferromagnetic ordering and paramagnetic contribution in Cu-Co-co-doped ZnO nanoparticles prepared by the hydrothermal method.

### 2.2. Solvothermal

Based on the hydrothermal method, the solvothermal method is based on the replacement of water with other solvents as a way to achieve the experimental purpose. The solvothermal method is favored by researchers because of its simple process, low equipment requirements, high purity, controllable particle size, and morphology of the prepared material [59]. In order to investigate the effect of mobile electrons on the magnetic properties of transition-metal-substituted ZnO nanocrystals, Anzelms Zukuls et al. [60] synthesized zinc acetate dihydrate (99.5%), manganese acetate tetrahydrate (99.0%), nickel acetate tetrahydrate (99.0%), and ferric chloride (98%); diolol^®^ (anhydrous ethanol denatured by addition of 2% isopropanol and 2% methyl ethyl ketone), 0.5 M anhydrous ethanol gallium chloride (99.99%) solution, and sodium hydroxide (98.0%) were used as the raw materials for the synthesis. Beginning with Solution A, 2 mmol of Me precursor salt was dissolved in 15 mL of ethanol (diol) and heated to 60 °C. In a two-necked flask with a round bottom, Solution B was made by combining 30 milliliters of ethanol (diol) with 0.6 g of NaOH, then heating it while stirring to reflux boiling. Next, using a syringe with a needle through a perforated rubber stopper, Solution A was introduced to Solution B. After 30 min of reflux stirring, the mixture was moved to an autoclave reactor vessel and heated to 150 °C for a whole day. After that, the autoclave was allowed to cool to ambient temperature. To remove by-products that included salt, the resulting nanocrystals were carefully cleaned three times using methanol and centrifugation. In the last stage, hexane was utilized for storage rather than methanol in order to prevent zinc oxide from degrading prematurely.

Also, Andris Šutka et al. [61] prepared dilute magnetic and plasma Co-Ga-co-doped ZnO nanocrystals by solvothermal synthesis in ethanol. When the co-dopant Ga^3+^ gradually replaces Zn^2+^ in Zn_0.95_Co_0.05_O, it appears that free electrons make it difficult for zincate ions to diffuse to the crystal surface due to Coulomb repulsion.

### 2.3. Sol-Gel

The sol-gel method has various advantages, such as a low cost, simple equipment, low operating temperature, and easy adjustment of composition and dopants [62,63]. Specifically, the sol-gel method has the following characteristics [64]: (i) the process is simple, the equipment requirements are low, and the material preparation process is easy to control, which allows for the preparation of some materials that cannot be prepared by traditional methods; (ii) the chemical composition of the material can also be precisely controlled, and it is easy to carry out precise doping, thus controlling its microstructure; (iii) the composition of the prepared material is uniform, and the concentration of the product is high; (iv) the sol-sintering temperature of the gel method is not very high and the film area formed on the substrate is large. The process of the sol-gel method can be divided into four steps: precursor dissolution, solution gelation, gel solidification, and solid sintering.

Abdullah S. Alshammari et al. [65] prepared co-doped ZnO nanostructured thin films of Cd and 1, 2, and 3 wt.% Na on a glass substrate by using sol-gel spin-coating technique. It was found that the band gap of the co-doped films increased with increasing Na concentration, and not only did the band gap of the co-doped nanostructured films change, but the grain size and morphology of the films were also considerably affected. Chien-Yie Tsay et al. [66] reported the preparation of Ga-N-co-doped ZnO thin films on alkali-free glass substrates using a sol-gel spin-coating process. The doping of Ga and N resulted in significant changes in the microstructure of the films, with a decrease in the roughness of the surface and an increase in the transparency in the visible range.

### 2.4. Other Methods

#### 2.4.1. Combustion

The combustion method is favored by many researchers for its advantages of a simple production process, fast reaction speed, high product purity, energy savings, and cost reduction [37,67,68]. P. Sathish et al. [69] prepared 2 at.% Ag (3, 6 at.%)-Fe-co-doped ZnO. With the increase in Fe doping concentration, the lattice parameters a and c marginally decreased as a result of the nanoparticles’ decreasing size. The decrease in the size of the nanoparticles was due to the increase in Fe doping concentration. The reduced crystal size meant that the Ag-Fe co-doping played a vital role in the study of improving the antimicrobial properties of ZnO nanopowders.

#### 2.4.2. Spray Pyrolysis

Spray pyrolysis is an ideal method for growing thin films because of the simple equipment required, low cost, easy doping of multiple elements, fast film growth rates, and short preparation cycles, and it also combines the features of both vapor- and liquid-phase methods for large-scale production. In addition, it provides an efficient method to grow and coat films using almost any element and does not require vacuum or high-quality targets or substrates [70,71,72,73]. B. Askri et al. [74] found that indium doping enhanced blue light emission intensity by increasing the carrier concentration at the oxygen vacancy level. Toshiyuki Fujimoto et al. [75] used an ultrasonic spray pyrolysis method. It was shown that ZnO particles loaded with 0.1% Au had the best photocatalytic activity, while ultrasonic spray pyrolysis could generate particles in one simple step.

#### 2.4.3. Wet Chemical “Liquid Ceramic”

B.B. Straumal et al. [76] prepared pure ZnO films using a wet chemical “liquid ceramic” method and concluded that the high-temperature ferromagnetic phenomenon of pure ZnO nanoparticles was often defect-driven, showing that the grain boundaries of ZnO ferromagnetic nanoparticle films prepared by the “liquid ceramic” method were surrounded by amorphous layers.

#### 2.4.4. Chemical

Eu-Ce-co-doped ZnO nanorods were successfully synthesized by a chemical precipitation method using polyvinylpyrrolidone as a surfactant by G. Murugadoss et al. [40]. The doping of Eu and Ce into the ZnO matrix caused the single-cell volume of the doped ZnO nanocrystals to expand, modulating the band gap of bulk ZnO (3.4 eV). It was shown that the product had good crystallinity and a nanoscale structure.

#### 2.4.5. RF Magnetron Sputtering

Liu et al. [41] found that the annealing temperature had a great influence on the crystal structure and optical properties of Al-Eu-doped ZnO films; thus, Al-Eu-doped ZnO annealed at 500 °C had good film quality.

#### 2.4.6. Acoustic Chemical

The acoustic chemical method provides ZnO nanoparticles with lamellar morphology. N.F. Andrade Neto et al. [42] prepared Co-Mn-co-doped ZnO nanoparticles by the acoustic chemical method. It was found that the ZnO powder did not form a secondary phase and had the initial morphology of a nanoplate. This morphology was lost after doping, forming small hemispherical nanoparticles, reducing the size of the ZnO nanoparticles and increasing their surface area.

#### 2.4.7. Electron Beam Evaporation Deposition

Li et al. [43] prepared Co-Cu-co-doped ZnO polycrystalline films on single-crystal Si (111) substrates using an electron beam evaporation deposition film-forming process. The Co-Cu co-doping of ZnO changed the forbidden bandwidth of ZnO, which promoted exciton complex luminescence. It was shown that a certain amount of Co-Cu co-doping affected the concentration of zinc vacancies and zinc gap-filling defects in the ZnO films, which resulted in a significant enhancement of the intensity of the violet, blue, and green peaks.

## 3. Performance Study of ZnO

### 3.1. Optical Performance

The presence of both substances in zinc oxide is also a good alternative to achieving optical stability [77]. In order to use ZnO as a photocatalyst in the visible region, the wide band gap of ZnO and its optical properties need to be reduced by techniques such as the introduction of appropriate dopants [78,79,80]. For example, the ionic radii of Ni^2+^ and Co^2+^ are comparable to those of Zn^2+^, so Lubna Mustafa et al. comfortably modulated the dopant ions at the host site, thus significantly affecting the optical properties without much impact on the structure [81]. In addition, Al-Eu-ZnO [82], V-La-ZnO [83], S-N-ZnO [84], Co-Ni-ZnO [24], etc. also effectively enhanced the optical properties of ZnO.

**Figure 2:**
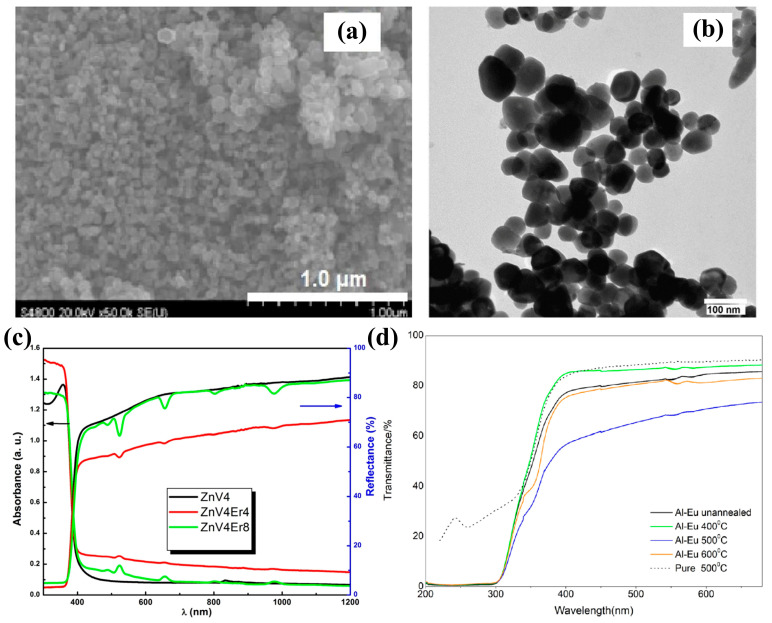
(**a**) SEM micrographs and (**b**) TEM micrographs of ZnV_4_Er_4_; (**c**) reflectance spectra measured at 300 K [87] (copyright © 2017, American Chemical Society); (**d**) relationship between transmittance and wavelength [41] (© 2021 Published by Elsevier B.V).

**Figure 3:**
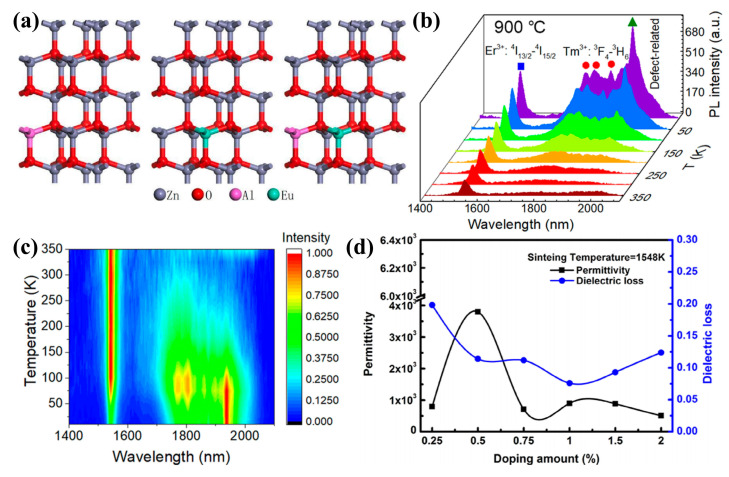
(**a**) The models of Al-, Eu-, and Al-Eu-doped ZnO [41] (© 2021 Published by Elsevier B.V.); (**b**) PL spectra of the Er-Tm-co-doped ZnO films annealed at 900 °C and measured at different temperatures in the range of 10–350 K (Er^3+^: ^4^I_13/2_–^4^I_15/2_ and Tm^3+^: ^3^F_4_–^3^H_6_: energy level jump process of Er^3+^ and Tm^3+^) and (**c**) temperature dependencies of the normalized PL intensity of the Er-Tm-co-doped ZnO films [90] (copyright © 2020, American Chemical Society); (**d**) dielectric properties of Zn_(1−2x)_(Li-In)_x_O samples sintered at 1548 K [92] (© 2016 Elsevier B.V. All rights reserved).

The co-dopant atoms and defects can effectively modulate the band gap of ZnO and will change the material properties in different ways, which is attractive for UV–visible technology [85,86]. J. El Ghoul [87] reported the structural and optical properties of V-Er-co-doped ZnO nanoparticles. The doping of Er and V ions in the ZnO crystal structure changed the morphology of the ZnO crystal structure. The structure is characterized as a fibrillated zincite structure with a grain size of about 45 nm, and secondary phases were detected. It has strong reflectivity in the visible range and high absorption in the UV spectral range (Figure 2a–c). The optical properties of ZnO nanostructures have attracted much attention due to the fascinating optoelectronic properties and the corresponding structural and dimensional diversity [88]. For example, Liu et al. [41] investigated the optical properties of Al-Eu-co-doped ZnO using first-principle and magnetron-sputtering techniques. Different models of Al, Eu, and Al-Eu-doped ZnO were constructed based on the energy principle. The transmittances of Al-Eu-co-doped ZnO films were all lower compared to pure ZnO, while the 500 °C-annealed Al-Eu-co-doped ZnO showed a lower transmittance in the wavelength range of 320~680 nm, indicating better absorption, which was in agreement with the simulation results (Figure 2d and Figure 3a).

The growing demand for information traffic urgently requires the development of light sources and optical amplifiers. Especially with the arrival of the 5G era, infrastructure development has put forward higher requirements for the entire optical communication industry. Traditional photodetectors may not be able to meet the requirements of certain special application scenarios due to their simple detection functions [89,90,91]. In 2020, Dong et al. [90] found that thermal treatment improves the quality of ZnO films and activates Tm^3+^ and Er^3+^ ions. Thereby, the emission lifetimes associated with rare earth ions increased significantly with the annealing temperature, and then the lifetimes and luminescence intensities increased significantly with the annealing temperature when the rare earth ions were directly excited. These results help to explore ways to increase Tm- and-Er related IR emission and point the way to the practical application of Er-Tm-co-doped ZnO thin films as photoemitters and IR broadband optical amplifiers (Figure 3b,c).

### 3.2. Electrical Performance

**Figure 4:**
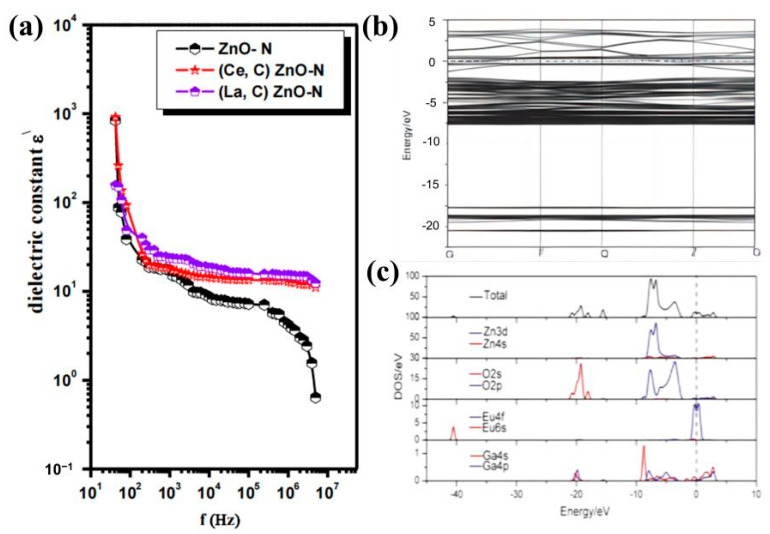
(**a**) Change in the dielectric constant with frequency for pure and co-doped ZnO samples [99] (© 2019 Elsevier Ltd. All rights reserved); (**b**) band structure and (**c**) density of states of GEZO [107] (© 2020 Elsevier B.V. All rights reserved).

The different behaviors (dielectric constant, modulus, conductivity, impedance, capacitance, etc.) of ZnO-based oxides have been extensively studied to understand the electrical properties of ZnO-based materials [93]. Ohmic and Schottky contacts have been demonstrated, but native point defects and surface chemistry strongly influence their properties [94]. For example, Li et al. [95] found that carriers were introduced in the ZnO crystal matrix due to the substitution of Gd^3+^ ions and Al^3+^ ions for Zn^2+^. As a result, the ZnO films have higher conductivity and carrier concentration with increasing Al doping. The high exciton binding energy is a special property of ZnO that is closely related to the dielectric constant of the material, and the eventual decrease in the dielectric constant increases the Coulomb interaction energy between electrons and holes, which may lead to an enhancement of the exciton binding energy [96]. The development of the dielectric properties is due to defects in the interstitial position of Zn excess and lack of oxidation, as pure ZnO is sensitive to oxidation and oxygen (O_2_) uptake tends to reduce its dielectric properties [97]. The enhanced dielectric properties of ZnO nanoparticles are due to (i) rotational directional polarization and space charge polarization due to a large number of oxygen vacancies and nano-size effects; (ii) the electronegativity of the doped atom being less than that of Zn; (iii) the movement of defect charges generating microcapacitance according to the grain boundary layer mechanism [92,97,98]. For example, Huang et al. [92] reported that Li-In-co-doped ZnO ceramics Zn_(1−2x)_(Li-In)_x_O obtained huge dielectric constants up to 3800 at an x of 0.5% (Figure 3d). The dielectric constant εʹ decreased with increasing application frequency, and remained constant at higher frequencies for the co-doped ZnO samples, while it decreased at higher frequencies for the pure ZnO samples. From Figure 4a, it can be seen that the relaxation process occurred in the dielectric constant εʹ of all ZnO samples [99].

Doping improves the electrical properties of ZnO, such as in Ag-Cu-co-doped ZnO, Mg-Al-co-doped ZnO, and Tb-Yb-co-doped ZnO [96,100,101,102,103,104,105], which have shown good results in terms of electrical properties. Various factors affect electrical conductivity, including the number of carriers, the mobility of free charges, and the availability of connected polar domains as conduction pathways [99]. In ZnO nanomaterials, depending on the synthesis process and post-annihilation treatment, native point defects such as zinc gaps and oxygen vacancies are induced as donors in the nanostructures, leading to different conduction mechanisms of free charge carriers and the formation of inhomogeneous dielectric structures [106]. Gao et al. [107] established a 2 × 2 × 2 super monolithic model of Ga-Eu-co-doped ZnO based on the density functional theory and investigated the energy band structure (Figure 4b) and density of states of Ga-Eu-co-doped ZnO structures (Figure 4c). The results showed that the energy band structure of the Ga-Eu-co-doped ZnO indicates that the electrons could move from the valence band to the conduction band more efficiently, which in turn led to the increase in the conductivity and the carrier concentration; the doped density of states shifted to the low-energy direction and the band gap became wider due to the dopant-generated carriers that changed the electronic state of ZnO.

### 3.3. Magnetic Properties

**Figure 5:**
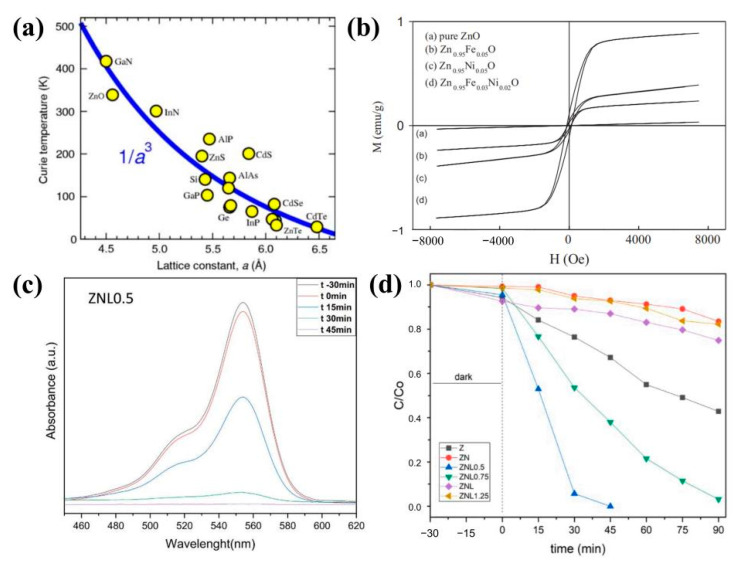
(**a**) Predicted Curie temperature as a function of lattice constant for a variety of semiconductors (S.C. Erwin (Naval Research Laboratory)). The materials predicted to have high Tc′s have large p-d hybridization and small spin–orbit interaction [111] (copyright © 2006, TMS); (**b**) M–H loops of Fe-and-Ni-doped ZnO samples [112] (copyright © 2014 Elsevier Ltd. and Techna Group s.r.l. All rights reserved); (**c**) time evolution of UV–Vis absorption spectra according to RhB degradation by ZNL0.5 and (**d**) RhB degradation rate during the photocatalytic process [151] (© 2023 Published by Elsevier Ltd.).

High magnetic fields have been used in the preparation of various materials for extreme conditions with high energy, indirect contact, and controllability. Similarly, in the preparation of ZnO-based dilute magnetic semiconductor materials, high magnetic fields are utilized to modulate the microstructure and magnetic properties, such as increasing the Curie temperature, inducing the transition from paramagnetic or antimagnetic to ferromagnetic, and increasing the ferromagnetism, as well as exploring the ferromagnetism mechanism from another angle [108]. Ferromagnetism is not an intrinsic property of the ZnO lattice, but of the ZnO/ZnO grain boundaries. Though the ZnO polycrystal can be converted to the ferromagnetic state even without doping with “magnetic atoms” such as Mn, Co, Fe, or Ni, this doping promotes the appearance of ZnO ferromagnetism, increases saturation magnetization, and decreases the amount of critical grain boundaries required for frequency modulation [109]. Co-doping affects the local structure and subsequent ferromagnetic ordering of transition-metal-doped ZnO [110]. The more traditional diluted magnetic semiconductor (DMS) materials, such as GaMnAs, InMnAs, and GaMnSb, show relatively low magnetic ordering temperatures (~170 K for GaMnAs). These traditional DMS materials provide a rich environment for the fundamental studies of semiconductor magnetism, but the lower Curie temperatures limit their potential applications. The wide-band materials GaMnN and ZnMnO promise strong room temperature ferromagnetism. Figure 5a shows that wider-band-gap semiconductors with smaller lattice constants, larger p-d hybridization, and smaller spin–orbit interactions are expected to have higher Curie temperatures [111]. Researchers have explained the observed magnetic properties through different mechanisms such as defects, secondary phases, TM clusters, impurity phases, bound magnetic polaritons, and vacancies [78]. For example, Wu et al. [112] used the hydrothermal method to synthesize the DMS Zn_0.95_Fe_0.05_Ni_0.05_O; Figure 5b shows the magnetic hysteresis loops of the Zn_0.95_Fe_0.05−x_Ni_x_O samples measured at room temperature. The results showed that the pure ZnO nanorods had minor paramagnetism at room temperature, while the Fe-Ni-co-doped ZnO exhibited significant ferromagnetism. The coercivity and saturation magnetization intensity of the co-doped samples were greater than those of the singly doped and pure ZnO samples.

For a clearer and more intuitive understanding, studies on the magnetic properties of co-doped ZnO are summarized in the table as shown in Table 1.

**Table 1:** Review of reported co-doped ZnO ferromagnets.

Composition	TM Content	Magnetism	T_C_ (K)	Fabrication Method	References
Fe-Nd-ZnO	Fe: 2.00% Nd: 1.00~5.00%	0.003 µ_B_	5–380	hydrothermal	[18]
Co-Ga-ZnO	Co: 5.00% Ga: 1.00%	−0.800 emu/g	500	PLD	[113]
Mn-P-ZnO	Mn: 0.05% P: 0.02%	0.050 emu/g	300	PLD	[114]
Co-Al-ZnO	Co: 0.04% Al: 0.01%	0.830 µ_B_/Co^2+^	5–350	molecular beam epitaxy	[115]
Mn-Ni-ZnO	Mn: 0.02% Ni: 0.01%	0.005 emu/g	50–350	hydrothermal	[19]
Na-Co-ZnO	Na: 0.03% Co: 0.05%	0.023 emu/g	300	sol-gel	[116]
Mn-Fe-ZnO	Mn: 0.02% Fe: 1.00%	~0.035 emu/g	2–350	in situ vapor-phase transport approach	[117]
Ag-N-ZnO	Ag: 3.00% N: 5.00%	2.300 emu/cm^3^	4300	RF sputtering	[118]
Fe-Mg-ZnO	Fe: 0.86% Mg: 0.04%	-	5–400	sol-gel	[119]
Mn-N-ZnO	Mn: 4.13% N: 1.88%	0.120 and 0.170 kA·m^−1^	300	sol-gel	[120]
Bi-Cu-ZnO	Bi: Below detection limit Cu: 0.60%	~0.500 emu/cm^3^	10–300	a vapor-phase transport	[121]
Cr-Co-ZnO	Cr: 0.09% Co: 0.03%	0.010 emu/g	10–300	citric gel route	[122]
Fe-Co-ZnO	Fe: 0.05% Co: 0.05%	-	5–300	sol-gel	[123]
Al-Mn-ZnO	Al: 0.03% Mn: 0.03%	0.019 emu/g	300–503	sol-gel	[124]
In-Mn-ZnO	In: 0.10% Mn: 0.10%	0.080 emu/g	2–300	solvothermal	[125]
Mn-Ni-ZnO	Mn: 0.04% Ni: 0.03%	0.015 × 10^−9^ emu/g	-	sol-gel	[126]
Na-F-ZnO	Na: 0.01% F: 0.01%	3.020 × 10^−4^ emu/g	-	sol-gel	[127]
Cr-Ni-ZnO	Cr: 1.00% Ni: 1.00%	0.010 emu/g	20–300	hydrothermal	[128]
Ni-Na-ZnO	Ni: 3.00% Na: 3.00%	0.160 emu/g	-	pulsed laser deposition	[129]
Cu-Co-ZnO	Cu: 0.02% Co: 0.02%	-	-	RF magnetron sputtering technique	[130]
Mn-Sn-ZnO	Mn: 3.00% Sn: 5.00%	6.000 × 10^−5^ emu	250	vapor transport	[131]
Nd-Mn-ZnO	Nd: 1.00% Mn: 1.00%	-	5–290	PLD	[132]
Mn-Na-ZnO	Mn: 0.05% Na: 0.05%	1.520 µ_B_	300	PLD	[133]
Co-Eu-ZnO	Co: 0.04% Eu: 0.04%	65.000 emu/cm^3^	77	ion implantation	[134]
F-Na-ZnO	F: 0.03% Na: 0.03%	0.053 emu/g	300	MEMS	[135]
Fe-Co-ZnO	Fe: 2.00% Co: 2.00%	0.960 emu/cm^3^	30–300	CVD	[136]
Li-Co-ZnO	Li: 0.10% Co: 0.05%	~0.480 µ_B_/Co	5–300	soft chemical	[137]
Co-Ga-ZnO	Co: 5.00% Ga: 1.00%	0.420 µ_B_/Co	300	PLD	[138]
Ni-Li-ZnO	Ni: 0.03% Li: 0.03%	0.800 emu/g	25–290	solvothermal	[139]
Cu-Al-ZnO	Cu: 0.02% Al: 3.00%	6.800 emu/cm^3^	5–300	PLD	[140]

### 3.4. Photocatalytic

Photocatalytic degradation is considered to be the best method for degrading organic waste pollutants without any toxic by-products compared to other conventional wastewater treatment methods due to its biological and chemical inertness, high capacity, cost-effectiveness, and long-term stability against photocorrosion and chemical corrosion [141,142,143]. Up to now, several oxide-based photocatalysts have been studied, including TiO_2_, ZnO, SnO_2_, Fe_2_O_3_, In_2_O_3_, and WO_3_ [144,145,146,147,148,149]. Among them, ZnO strongly supports the photocatalytic degradation of dyes, even though doping ZnO can improve the degradation efficiency [150]. Recently, A. Ferreiro et al. [151] investigated the photocatalytic properties of Nd-Li-co-doped ZnO nanoparticles synthesized by the polyol method, and the highest photocatalytic activity of the ZNL0.5 samples in rhodamine B (2.5 ppm) solution was due to the synergistic effect of the energy levels close to the conduction bands and the higher specific surface area (Figure 5c,d).

**Figure 6:**
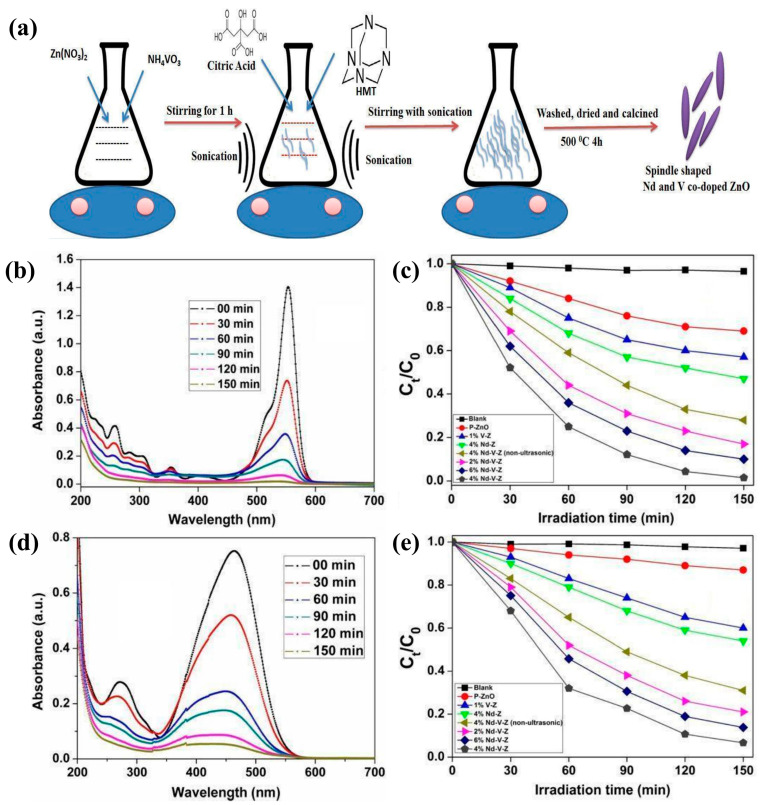
(**a**) Schematic illustration of the synthesis of Nd-and-V-co-doped ZnO; change in absorption spectra of RhB (**b**) and change in concentration of RhB as a function of time in the absence and presence of pure ZnO and different doped samples under visible light irradiation in (**c**); change in absorption spectra of MO (**d**) and change in concentration of MO as a function of irradiation time in the absence and presence of pure ZnO and different doped samples under visible light irradiation in (**e**) [152] (© 2018 Elsevier B.V. All rights reserved).

**Figure 7:**
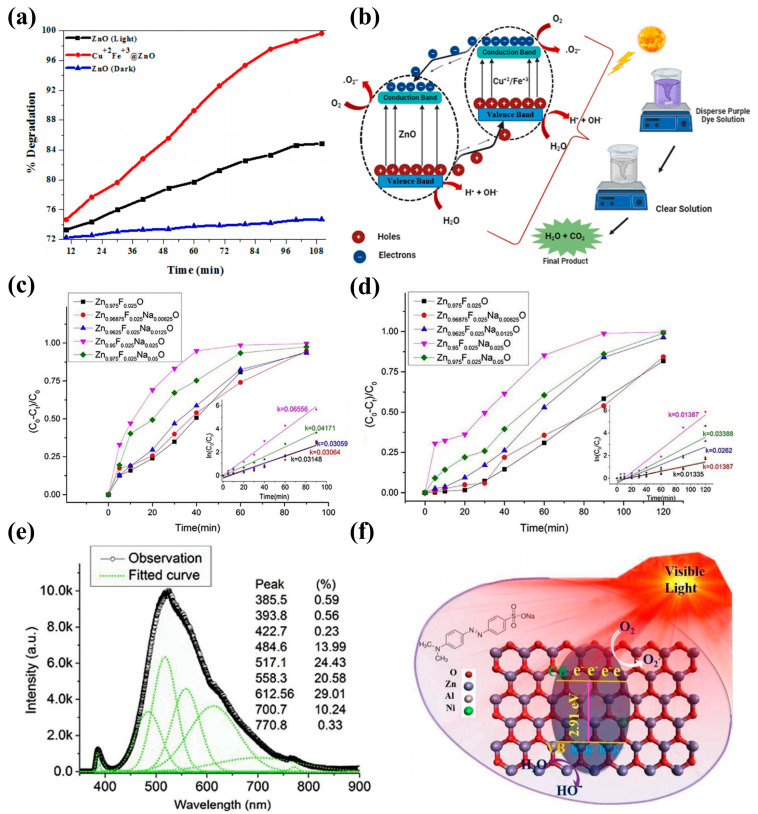
(**a**) Percentage degradation and (**b**) hypothesized mechanism for photocatalytic degradation of disperse purple dyes [153] (© 2024 Elsevier B.V. All rights reserved); photodegradation of MB with Zn_0.975−x_Na_x_F_0.025_O photocatalysts under (**c**) simulated sunlight irradiation and (**d**) UV light irradiation. The insets are the corresponding plots of ln (C_0_/C) versus irradiation time for MB photodegradation. (**e**) Gaussian deconvolution of PL spectra of Zn_0.975−0.025_Na_0.025_F_0.025_O nanocrystals [154] (© 2019 Elsevier Ltd. and Techna Group s.r.l. All rights reserved); (**f**) potential catalytic mechanism for Al-Ni-co-doped ZnO in organic dye degradation [155] (© 2018 Elsevier B.V. All rights reserved).

ZnO nanostructures are potential candidates for water treatment. Umair Alam et al. developed an ultrasound-assisted sol-gel method to synthesize spindle-shaped Nd-V-co-doped ZnO for the reduction of organic pollutants (Figure 6a). The results showed that the photocatalytic activity of co-doped ZnO was more significant than that of mono- and undoped ZnO. In addition, 4% Nd-V-co-doped ZnO showed excellent performance in degrading methyl orange and RhB due to its effective carrier separation and extended light absorption (Figure 6b–e) [152]. The simultaneous doping of two metals in oxide semiconductor materials is considered an effective strategy for mitigating carrier complexation and enhancing photocatalytic activity. Under UV light, Muhammad Mubeen Tahir et al. [153] investigated the rate at which Cu^+2^/Fe^+3^-co-doped zinc oxide degraded in dye solutions. Moreover, based on dye degradation, the reaction kinetics and possible reaction mechanisms were investigated. The findings demonstrated that the dye degradation rate and photocatalytic activity were increased by the doping of Cu^+2^ and Fe^+3^ (Figure 7a,b).

For a clearer and more intuitive understanding, studies on the photocatalytic properties of co-doped ZnO are summarized as shown in Table 2.

**Table 2:** A review of the main reported photocatalytic properties of ZnO.

Composition	Light Source	Pollutant	Experimental Conditions	PE	Fabrication Method	References
Fe-Cu-ZnO/GO	UV	Dark green dye	CL = 0.05 g·L^−1^ t_r_ = 90 min	99.28%	sol-gel	[156]
C-Ce-ZnO/ C-La-ZnO	visible	MB	CL = 0.01 g·L^−1^ t_r_ = 80 min	89%/ 99%	sol-gel	[99]
Fe-Pb-ZnO	UV	MB	t_r_ = 90 min	reduced	microwave-assisted hydrothermal	[157]
Fe-Eu-ZnO	solar light	MO	CL = 0.001 g·L^−1^ t_r_ = 120 min	94%	co-precipitation	[158]
Al-Er-ZnO	450 W Xe arc lamp	RhB	t_r_ = 120 min	above 90%	hydrothermal	[159]
Ni-Co-ZnO	100 W tungsten lamp	RhB	t_r_ = 360 min	42%	co-precipitation	[13]
Ag-Al-ZnO	UV	MB	t_r_ = 120 min	57%	microwave-assisted chemical synthesis technique	[150]
Mn-Cu-ZnO	UV	MB	t_r_ = 30 min	-	hydrothermal	[160]
La-Ce-ZnO	UV	MB	CL = 0.01 g·L^−1^ t_r_ = 120 min	95.2%	Solvothermal route.	[161]
Cr-In-ZnO	visible	MB	t_r_ = 180 min	95%	Spray pyrolysis technique	[162]
In-Mg-ZnO	UV	OR-II	t_r_ = 240 min	88.57%	chemical co-precipitation	[28]
Ce-Ni-ZnO	UV	MB	t_r_ = 120 min	81.3%	sol-gel	[29]
Er-Al-ZnO	UV	RhB	t_r_ = 120 min	93%	hydrothermal	[163]
Eu-Tb-ZnO	UV	MB	t_r_ = 50 min	99.9%	combustion	[141]
Bi-N-ZnO	UV	RhB	t_r_ = 180 min	89%	hydrothermal	[164]
Gd-N-ZnO	UV	MB	t_r_ = 60 min	87%	wet chemical co-precipitation	[142]
Ag-N-ZnO	visible	MO	t_r_ = 120 min	98.82%	sol-gel	[36]

Note. PE = photodegradation efficiency. CL = catalyst loading. t_r_ = irradiation time.

In summary, the dopant had little impact on the structural integrity, and improving dopant concentrations caused the ZnO nanorods’ diameter to grow but their density to decrease. Furthermore, it was shown that when the dopant concentration increased, optical transmittance often increased as well. The production of tailored nanomaterials appropriate for a range of applications is made possible by the interplay of growth temperature, dopant type, and concentration in adjusting the structural, morphological, optical, electrical, magnetic, and photocatalytic features of ion-doped ZnO nanomaterials [165,166,167,168].

## 4. Application of ZnO Nanomaterials

ZnO is an adaptable material with properties such as a large specific area, non-toxicity, good compatibility, and high isoelectric point, and these excellent properties represent a wide range of applications for ZnO materials in many fields [169,170].

### 4.1. Photocatalyst

The applications of the catalysis of ZnO are mainly concerned with green energy and environmental issues such as CO_2_ hydrogenation to fuel, methanol steam reforming to hydrogen, biodiesel production, and the photodegradation of pollutants [171]. MB dye is one of the most common organic pollutants in wastewater compared to all industrial wastewater and other textiles and is harmful to the human body as it causes vomiting, cyanosis, increased heart rate, skin diseases, and intestinal problems. Therefore, the appropriate techniques to degrade the toxic organic compounds in MB dyes have received the attention of a wide range of researchers to provide an efficient and green solution to environmental problems [172,173]. For example, Yuan et al. [154] prepared Na-F-co-doped ZnO nanocrystals by a modified polymer network gel method and investigated their photocatalytic activity and defect-related photoluminescence at room temperature. It was shown that Na-F-co-doped ZnO photocatalysts with different Na concentrations improved the photocatalytic degradation efficiency of MB under UV irradiation and simulated sunlight. Zn_0.95_Na_0.025_F_0.025_O induced the complete decomposition (more than 90%) of MB (4 mg/L) in water after 40 min. The fluorescence intensity and photocatalytic activity increased with the increasing concentration of Na doping, introducing receptor-related defects. In practical applications, the PL test is a powerful means to quickly and concisely evaluate the photocatalytic activity of ZnO-based materials (Figure 7c–e).

**Figure 8:**
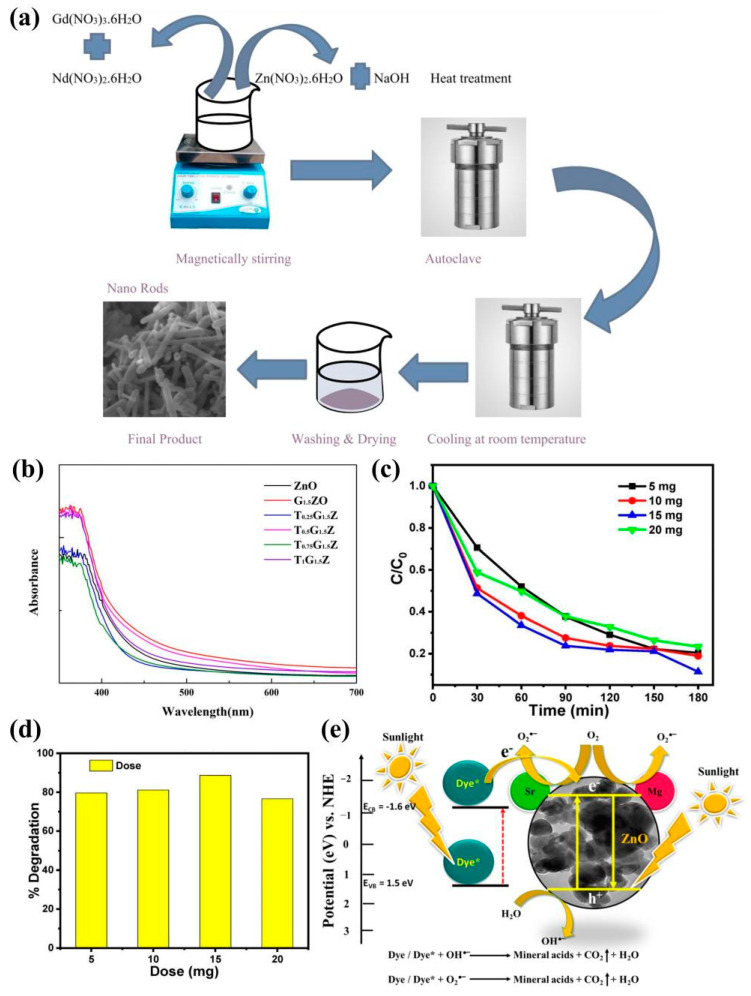
(**a**) Schematic diagram for the preparation of Nd-Gd-co-doped ZnO by hydrothermal method [174] (© 2020 Elsevier Ltd. and Techna Group s.r.l. All rights reserved); (**b**) UV–vis–NIR spectra of ZnO, GZO, and T_x_G_1.5_Z nanopowders [175] (copyright © 2022, the author(s), under exclusive license to Springer Science Business Media, LLC, part of Springer Nature); (**c**) the plots of ln(C_0_/C) vs. reaction time for the photocatalytic degradation of LO and (**d**) the degradation efficiency of various doses of 0.06 M Mg-Sr-co-doped ZnO nanocrystals, and (**e**) improved photocatalytic degradation mechanism of 0.06 M Mg-Sr-co-doped ZnO in the presence of sunlight [176] (© 2023 Copyright Clearance Center, Inc. All rights reserved).

Photocatalysis is applied to many current environmental problems. Some researchers proposed a possible photocatalytic degradation mechanism by performing free-radical-scavenging experiments and explained the photocatalytic activity of the Al-Ni-co-doped ZnO photocatalyst synthesized by the high-energy ball-milling method (Figure 7f) [155]. The photocatalytic degradation efficiency of the Nd-Gd-co-doped ZnO samples was improved compared with that of pure ZnO. The same process can be repeated for all Nd-Gd-co-doped ZnO samples by simply changing the doping concentration/concentration of the precursor solution (Figure 8a) [174]. When the doping concentration is low, Ga and Ti can replace the vacancies of Zn. As a result, the particle size of ZnO decreases, then the absorption of visible light is enhanced, and then the catalytic degradation ability is significantly improved (Figure 8b) [175]. Alkaline earth metal doping is more effective than transition metal doping in reducing the optical threshold energy of semiconductors. Sanakousar F. M. et al. [176] synthesized Mg-Sr-co-doped ZnO nanocrystals (0.03, 0.06, and 0.09 M Mg) by a simple co-precipitation method and used them for the photocatalytic degradation of leuko-olive reactive dyes. It was further demonstrated that the 0.06 M Mg-Sr-co-doped ZnO photocatalyst was the most cost-effective, in an attempt to better understand the charge transfer mechanism and to investigate the band edge positions of the 0.06 Mg-Sr-co-doped ZnO (Figure 8c–e).

### 4.2. Solar Cells

**Figure 9:**
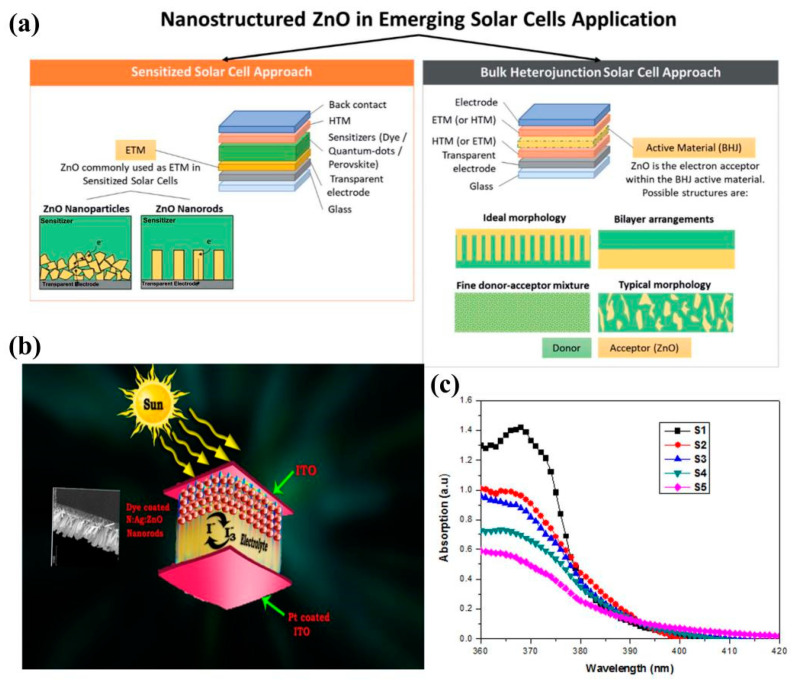
(**a**) Nanostructured ZnO in emerging solar cell applications [180]; (**b**) graphical abstract; (**c**) UV–Vis graph of undoped, N-doped, and Ag-co-doped ZnO nanostructure samples [183] (copyright © 2019, Springer Science Business Media, LLC, part of Springer Nature).

In order to protect the atmosphere and reduce carbon emissions, fossil fuels urgently need to utilize solar energy as a carbon-free energy source to solve the problems of the energy crisis, environmental pollution, and global warming [177,178]. Many researchers have utilized the photovoltaic effect to convert sunlight into electricity, and various types of solar cells have been developed due to their cleanliness, sustainability, and renewability [179,180,181]. Conversion efficiency (η) is the most important parameter for evaluating the performance of a solar cell and indicates the extent to which incident solar energy is converted into maximum output power [177]. Losses in the energy conversion process of solar cells are reduced by up-conversion (UC), down-conversion (DC), and down-shift (DS) [182]. ZnO is considered one of the potential materials for solar cell applications due to its high electrical conductivity, electron mobility, stability against photocorrosion, and low-cost availability. Therefore, ZnO has many applications in emerging solar cells (Figure 9a), such as dye-sensitized solar cells (DSSCs), chalcogenide sensitized solar cells (PSCs), perovskite solar cells (PVSCs), etc. [180].

**Figure 10:**
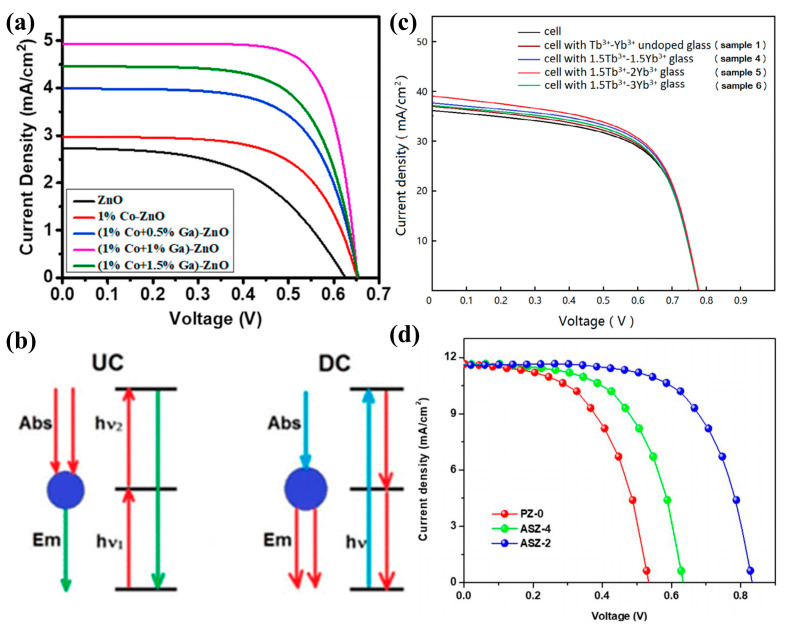
(**a**) J–V curve of undoped ZnO and Ga-Co-co-doped ZnO-based DSSCs [185] (© 2020 Elsevier Ltd. and Techna Group S.r.l. All rights reserved); (**b**) simplified energy-level diagrams for UC and DC processes [182] (copyright © 2021, American Chemical Society); (**c**) I–V curves of solar cells only and solar cells with the glass samples [187] (© 2019 Published by Elsevier B.V.); (**d**) J–V characterizations of PZ, ASZ-2, and ASZ-4 [188] (copyright © 2023, the author(s), under exclusive license to Springer Science Business Media, LLC, part of Springer Nature).

The low photovoltaic performance of DSSCs based on ZnO-based photoelectrodes is due to the fast compounding rate and narrow absorption spectral range. By doping modified ZnO, the new energy levels formed in its d-orbitals can improve the optical and electrical properties of ZnO by changing the band gap [184]. In 2019, researchers reported the preparation of N-Ag-co-doped ZnO nanorod structures and their photovoltaic applications. The synthesized N-Ag-co-doped ZnO nanorods were used as photoanodes for dye-sensitized solar cells (DSSCs). The efficiency of the photoanode DSSC-co-doped sample (S5) prepared under a 100 mW/cm^2^ light source was significantly improved by 5.105% (0.707%) as compared to the undoped ZnO (S1) (Figure 9b,c) [183]. Moreover, the DSSCs formed by ZnO-based photoanodes co-doped with 1% Co and 1% Ga showed more than 100% efficiency over pure ZnO-based cells (Figure 10a) [185].

The UC process is often referred to as a non-linear anti-Stokes process in which two or more low-energy photons (near-infrared region) are absorbed and one high-energy photon (UV–visible region) is emitted [182]. At the same time, DC is the exact opposite process of UC (Figure 10b). The DC process is used to modify the incident solar radiation in order to reduce the power loss of the solar cell [186]. The researchers found that the Tb-Yb-co-doped ZnO solar conversion efficiencies increased by 4.98%, 8.60%, and 3.68%, respectively, with increasing Yb^3+^ (1.5, 2, 3 mol%) concentration (Figure 10c) [187]. A. Pramothkumar et al. [188] increased the optical absorbance and band gap position by introducing Al-Sn (2 wt.% and 4 wt.%) as a co-dopant, thereby lowering the energy barrier during electron extraction. It also hinders the interfacial complex loss between the electron transport layer/calcite. The Al-Sn (2 wt.%)-co-doped ZnO had better power conversion efficiency, which was maintained at 77.37% compared to the other layers (Figure 10d).

### 4.3. Gas Sensors

**Figure 11:**
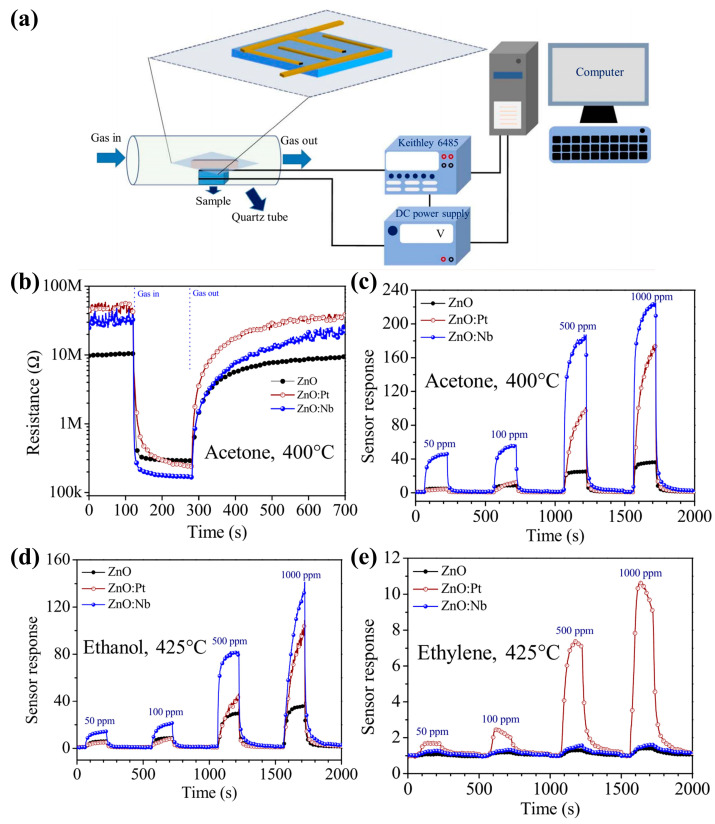
(**a**) The schematic diagram of gas sensing measurement; (**b**) dynamic resistance response characteristics of ZnO, ZnO:Pt, and ZnO:Nb sensors upon exposure to 1000 ppm acetone concentration at an operating temperature of 400 °C, and dynamic sensor response versus time, dependent on exposure to (**c**) acetone, (**d**) ethanol, and (**e**) ethylene with concentrations ranging from 50 to 1000 ppm at the optimum operating temperatures [49] (© 2017 Elsevier Ltd. and Techna Group s.r.l. All rights reserved).

In recent years, gas sensors have received a lot of attention due to their important role in atmospheric environmental monitoring, medical diagnostics, volatile organic compounds (VOCs) and toxic gases, etc. Importantly, ZnO exhibits an abundance of interesting nanostructures that have demonstrated the potential to achieve highly sensitive gas sensors. Many factors affect the performance of ZnO material transducers, including various structural and performance parameters. Structural parameters mainly include morphology, size, and porosity. In addition, some researchers proposed a highly selective acetone gas sensor based on ZnO nanostructures coated with Pt and Nb using the DC pulse sputtering technique. The gas-sensitive properties of acetone, ethanol, and ethylene vapors were evaluated at the operating temperatures of 275~450 °C. Meanwhile, the gas-sensitive measurement principle was explored (Figure 11a–e) [44,45,46,47,48,49].

**Figure 12:**
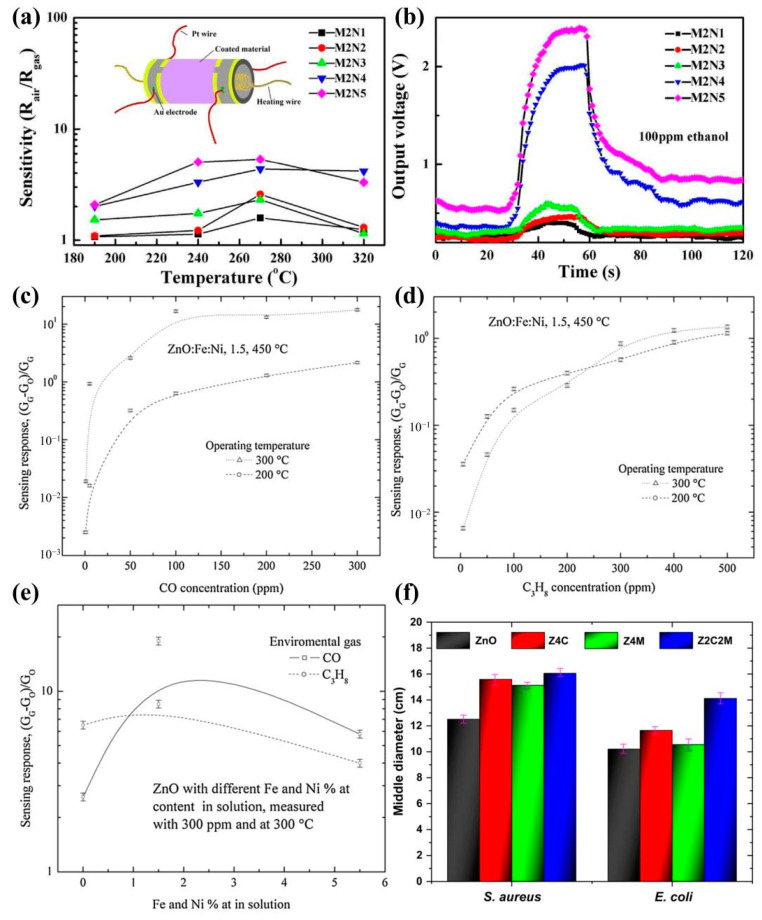
(**a**) Sensor sensitivity vs. operating temperature curves and (**b**) plot of change in output voltage of Mn-Ni-co-doped ZnO NR gas sensors for different Ni concentrations [19] (copyright © 2019, Elsevier); effect of carbon monoxide (**c**) and propane (**d**) concentration on sensing response of ZnO films doped with 1.5 at.% Fe and Ni measured at 200 and 300 °C; (**e**) maximum sensing response of all ZnO films measured at 300 ppm and 300 °C [189] (copyright © 2020, Springer Science Business Media, LLC, part of Springer Nature); (**f**) inhibition halos against *S. aureus* and *E. coli* bacteria for the ZnO samples [42] (copyright © 2019, The Minerals, Metals & Materials Society).

Since gas sensitivity is a very important property of ZnO materials, Gao et al. [19] fabricated Mn-Ni-co-doped ZnO NR as a gas sensor and measured its gas sensitivity. Figure 12a shows the gas-sensing response of the Mn-Ni-co-doped ZnO sensor for 100 ppm ethanol at different operating temperatures. The gas responses of the Mn-Ni-co-doped ZnO NRs all reached a maximum at 270 °C. Figure 12b shows the real-time response curves of the Mn-Ni-co-doped ZnO NR sensors at 270 °C and 100 ppm ethanol atmosphere. On the one hand, Mn-Ni co-doping improved the performance of the ZnO sensors because transition metal ion doping affected the defect state and microstructure of the ZnO nanorods; on the other hand, Mn and Ni co-doping improved the performance of the ZnO sensors due to the presence of effective surface-active sites for oxygen adsorption onto oxygen vacancies.

V. K. Jayaraman et al. [189] successfully prepared Fe-Ni-co-doped ZnO thin films using the ultrasonic chemical spraying technique. For the co-doped ZnO films, the corresponding diffraction peaks coincided with the hexagonal shape of fibrous zincite. The surface morphology of the co-doped ZnO films changed significantly with the doping level. The gas-sensing response of the Fe-Ni-co-doped ZnO films was tested in carbon monoxide (Figure 12c) and propane (Figure 12d) gases and was found to be more pronounced at higher concentrations than at lower concentrations. Figure 12e shows the maximum value of the sensing response measured at 300 °C and a gas concentration of 300 ppm for CO and C_3_H_8_. The results showed that Fe-Ni-co-doped ZnO films helped to improve the sensing response up to less than 5%.

### 4.4. Biomedicine

The deteriorating environment and rapidly aging population, leading to increased diseases, health care issues, and medical costs, especially in developing countries, have led to a high demand for better and lower-cost biomedical devices with novel biological functions. Nanoparticles have been continuously evaluated and used in many industrial applications. In particular, ZnO has received much attention due to its UV-filtering, anti-inflammatory (medicine), antifungal, high catalytic, and antibacterial activities [190,191,192].

N.F. Andrade Neto et al. [42] prepared Co-Mn-co-doped ZnO nanoparticles using the sonochemical method. The results showed that the inhibitory effect of the co-doped ZnO nanoparticles on *Escherichia coli* (Gram-negative) and *Staphylococcus aureus* (Gram-positive) was increased by 4 cm and 3 cm, respectively, as compared to pure ZnO (Figure 12f).

Above all, the availability of the wide band gap and the large exciton binding energy are excellent characteristics of ZnO nanomaterials, but there are some limitations, which can be improved by co-doping techniques. Due to the versatility of their synthesis methods, ZnO nanostructures of various morphologies such as nanorods and nanoflowers can be synthesized. Therefore, the morphology and properties of co-doped ZnO nanostructures are of significant importance for potential applications.

## 5. Conclusions

ZnO is an adaptable material with the advantage of a wide band gap, large exciton binding energy, high sensitivity, large specific area, and non-toxicity. The performance of ZnO nanomaterials has been continuously improved by co-doping due to their excellent properties in photocatalysis, solar cells, gas sensors, biomedicine, and other fields of application. As a promising semiconductor material, although some breakthroughs have been made in some fields, challenges and opportunities also exist, and new preparation methods, precise characterization techniques, and key factors for computational simulation still need to be further studied.

## Data Availability

No new data were created or analyzed in this study.
